# *N*-(2,3-Dimeth­oxy­benzyl­idene)naphthalen-1-amine

**DOI:** 10.1107/S1600536811000079

**Published:** 2011-01-08

**Authors:** Ailing Guo, Shurong Zhang, Xiaofang Liu, Jianhua Jiao

**Affiliations:** a Department of Traditional Chinese Pharmacology, Shanxi University of Traditional Chinese Medicine, Taiyuan 030024, People’s Republic of China

## Abstract

The title compound, C_19_H_17_NO_2_, represents a *trans* isomer with respect to the C=N bond. The dihedral angle between the planes of the naphthyl ring system and the benzene ring is 71.70 (3)°. In the crystal, weak C—H⋯O hydrogen bonding is present.

## Related literature

For properties of Schiff bases, see: Chen *et al.* (2008 ▶); May *et al.* (2004 ▶); Weber *et al.* (2007 ▶). For related structures, see: Tariq *et al.* (2010 ▶); Zhu *et al.* (2010 ▶).
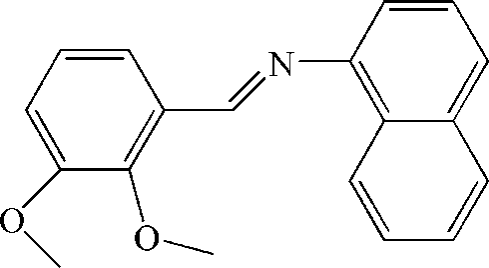


## Experimental

### 

#### Crystal data

C_19_H_17_NO_2_
*M* *_r_* = 291.34
Orthorhombic, 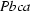

*a* = 7.7163 (7) Å
*b* = 17.0786 (16) Å
*c* = 23.427 (2) Å
*V* = 3087.3 (5) Å^3^
*Z* = 8
Mo *K*α radiation
μ = 0.08 mm^−1^
*T* = 298 K
0.48 × 0.45 × 0.36 mm

#### Data collection

Bruker SMART CCD area-detector diffractometer
Absorption correction: multi-scan (*SADABS*; Bruker, 2007 ▶) *T*_min_ = 0.962, *T*_max_ = 0.971
14855 measured reflections
2721 independent reflections
1452 reflections with *I* > 2σ(*I*)
*R*_int_ = 0.057

#### Refinement

*R*[*F* ^2^ > 2σ(*F* ^2^)] = 0.048
*wR*(*F* ^2^) = 0.187
*S* = 1.12
2721 reflections
202 parameters
H-atom parameters constrained
Δρ_max_ = 0.18 e Å^−3^
Δρ_min_ = −0.15 e Å^−3^

### 

Data collection: *SMART* (Bruker, 2007 ▶); cell refinement: *SAINT* (Bruker, 2007 ▶); data reduction: *SAINT*; program(s) used to solve structure: *SHELXS97* (Sheldrick, 2008 ▶); program(s) used to refine structure: *SHELXL97* (Sheldrick, 2008 ▶); molecular graphics: *SHELXTL* (Sheldrick, 2008 ▶); software used to prepare material for publication: *SHELXTL*.

## Supplementary Material

Crystal structure: contains datablocks I, global. DOI: 10.1107/S1600536811000079/pv2375sup1.cif

Structure factors: contains datablocks I. DOI: 10.1107/S1600536811000079/pv2375Isup2.hkl

Additional supplementary materials:  crystallographic information; 3D view; checkCIF report

## Figures and Tables

**Table 1:** Hydrogen-bond geometry (Å, °)

*D*—H⋯*A*	*D*—H	H⋯*A*	*D*⋯*A*	*D*—H⋯*A*
C8—H8*C*⋯O1^i^	0.96	2.54	3.232 (4)	129

Symmetry code: (i) 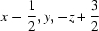.

**Fig. 1.**
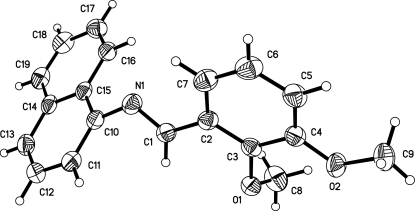
A view of the molecular structure of the title compound; displacement ellipsoids are drawn at the 30% probability level.

## supplementary crystallographic information

### Comment

The Schiff bases have received considerable attention for many years, primarily due to their importance as ligands in metal complexes with special magnetic (Weber *et al.*, 2007), catalytic (Chen *et al.*, 2008) and biological properties (May *et al.*, 2004). Here, we report the crystal structure of the title compound.

The title molecule (Fig. 1) represents a *trans*-isomer with respect to the C11═N1 bond. The planes of the aromatic systems of the the naphthyl and benzene groups, C10—C19 and C2—C7, respectively, form dihedral angle of 71.70 (3)°. The bond distances and bond angles in the title compound are in agreement with the corresponding bond distances and angles reported in the crystale structures of closely related compounds, (Tariq *et al.*, 2010; Zhu *et al.*, 2010). The crystal structure of the title compound displays weak intermolecular interactions C8—H8C···O1 as well as intramolecular hydrogen bonds, C8—H8C···O2 and C16—H16···N1.

### Experimental

1-Naphthylamine (0.72 g, 5 mmol) and 2,3-dimethoxybenzaldehyde (0.83 g, 5 mmol) were dissolved in ethanol (20 ml). The mixture was refluxed for 2 h, and then cooled to room temperature. The reaction mixture was filtered and the filtered cake was recreystallized from ethyl alcohol (yield 80%). Crystals of the title compound suitable for X-ray diffraction were obtained by slow evaporation of an ethanol solution.

### Refinement

H atoms were placed in idealized positions and allowed to ride on their respective parent atoms, with C—H = 0.93–0.96 Å and *U*_iso_(H) = 1.2 or 1.5 times *U*_eq_(C).

### Crystal data

**Table:**

C_19_H_17_NO_2_	*F*(000) = 1232
*M**_r_* = 291.34	*D*_x_ = 1.254 Mg m^−^^3^
Orthorhombic, *P**b**c**a*	Mo *K*α radiation, λ = 0.71073 Å
Hall symbol: -P 2ac 2ab	Cell parameters from 2315 reflections
*a* = 7.7163 (7) Å	θ = 2.4–24.2°
*b* = 17.0786 (16) Å	µ = 0.08 mm^−^^1^
*c* = 23.427 (2) Å	*T* = 298 K
*V* = 3087.3 (5) Å^3^	Block, colorless
*Z* = 8	0.48 × 0.45 × 0.36 mm

### Data collection

**Table:**

Bruker SMART CCD area-detector diffractometer	2721 independent reflections
Radiation source: fine-focus sealed tube	1452 reflections with *I* > 2σ(*I*)
graphite	*R*_int_ = 0.057
φ and ω scans	θ_max_ = 25.0°, θ_min_ = 2.4°
Absorption correction: multi-scan (*SADABS*; Bruker, 2007)	*h* = −8→9
*T*_min_ = 0.962, *T*_max_ = 0.971	*k* = −20→10
14855 measured reflections	*l* = −27→27

### Refinement

**Table:**

Refinement on *F*^2^	Secondary atom site location: difference Fourier map
Least-squares matrix: full	Hydrogen site location: inferred from neighbouring sites
*R*[*F*^2^ > 2σ(*F*^2^)] = 0.048	H-atom parameters constrained
*wR*(*F*^2^) = 0.187	*w* = 1/[σ^2^(*F*_o_^2^) + (0.0462*P*)^2^ + 2.0418*P*] where *P* = (*F*_o_^2^ + 2*F*_c_^2^)/3
*S* = 1.12	(Δ/σ)_max_ < 0.001
2721 reflections	Δρ_max_ = 0.18 e Å^−^^3^
202 parameters	Δρ_min_ = −0.15 e Å^−^^3^
0 restraints	Extinction correction: *SHELXL97* (Sheldrick, 2008), Fc^*^=kFc[1+0.001xFc^2^λ^3^/sin(2θ)]^-1/4^
Primary atom site location: structure-invariant direct methods	Extinction coefficient: 0.0059 (11)

### Special details

**Table:**

Geometry. All e.s.d.'s (except the e.s.d. in the dihedral angle between two l.s. planes) are estimated using the full covariance matrix. The cell e.s.d.'s are taken into account individually in the estimation of e.s.d.'s in distances, angles and torsion angles; correlations between e.s.d.'s in cell parameters are only used when they are defined by crystal symmetry. An approximate (isotropic) treatment of cell e.s.d.'s is used for estimating e.s.d.'s involving l.s. planes.
Refinement. Refinement of *F*^2^ against ALL reflections. The weighted *R*-factor *wR* and goodness of fit *S* are based on *F*^2^, conventional *R*-factors *R* are based on *F*, with *F* set to zero for negative *F*^2^. The threshold expression of *F*^2^ > σ(*F*^2^) is used only for calculating *R*-factors(gt) *etc*. and is not relevant to the choice of reflections for refinement. *R*-factors based on *F*^2^ are statistically about twice as large as those based on *F*, and *R*- factors based on ALL data will be even larger.

### Fractional atomic coordinates and isotropic or equivalent isotropic displacement parameters (Å^2^)

**Table:**

	*x*	*y*	*z*	*U*_iso_*/*U*_eq_	
N1	0.3928 (4)	0.08249 (16)	0.55054 (11)	0.0578 (8)	
O1	0.4329 (3)	0.18230 (12)	0.70513 (9)	0.0535 (6)	
O2	0.4198 (3)	0.10066 (14)	0.80315 (9)	0.0682 (7)	
C1	0.4312 (4)	0.11133 (19)	0.59845 (13)	0.0508 (8)	
H1	0.4599	0.1641	0.6006	0.061*	
C2	0.4323 (4)	0.06453 (19)	0.65073 (13)	0.0492 (8)	
C3	0.4280 (4)	0.10162 (18)	0.70328 (13)	0.0471 (8)	
C4	0.4265 (4)	0.05837 (19)	0.75393 (14)	0.0523 (8)	
C5	0.4328 (5)	−0.0221 (2)	0.75084 (16)	0.0660 (10)	
H5	0.4311	−0.0517	0.7842	0.079*	
C6	0.4417 (5)	−0.0591 (2)	0.69855 (17)	0.0714 (11)	
H6	0.4489	−0.1134	0.6971	0.086*	
C7	0.4402 (4)	−0.0173 (2)	0.64880 (16)	0.0624 (10)	
H7	0.4443	−0.0430	0.6139	0.075*	
C8	0.2786 (5)	0.2204 (2)	0.72204 (17)	0.0784 (12)	
H8A	0.1901	0.2114	0.6940	0.118*	
H8B	0.2996	0.2756	0.7254	0.118*	
H8C	0.2414	0.2001	0.7582	0.118*	
C9	0.4279 (6)	0.0592 (2)	0.85555 (14)	0.0817 (13)	
H9A	0.3322	0.0235	0.8579	0.122*	
H9B	0.4222	0.0956	0.8867	0.122*	
H9C	0.5347	0.0306	0.8575	0.122*	
C10	0.4014 (4)	0.13165 (18)	0.50188 (13)	0.0522 (8)	
C11	0.5459 (5)	0.1750 (2)	0.48899 (15)	0.0650 (10)	
H11	0.6406	0.1746	0.5136	0.078*	
C12	0.5514 (5)	0.2198 (2)	0.43916 (16)	0.0720 (11)	
H12	0.6505	0.2486	0.4308	0.086*	
C13	0.4152 (5)	0.2221 (2)	0.40291 (15)	0.0657 (10)	
H13	0.4217	0.2522	0.3699	0.079*	
C14	0.2641 (5)	0.17926 (18)	0.41459 (13)	0.0519 (8)	
C15	0.2558 (4)	0.13319 (17)	0.46482 (12)	0.0478 (8)	
C16	0.1014 (5)	0.09268 (19)	0.47708 (14)	0.0556 (9)	
H16	0.0948	0.0620	0.5098	0.067*	
C17	−0.0383 (5)	0.0976 (2)	0.44183 (15)	0.0644 (10)	
H17	−0.1399	0.0711	0.4509	0.077*	
C18	−0.0290 (5)	0.1425 (2)	0.39210 (16)	0.0697 (11)	
H18	−0.1246	0.1454	0.3680	0.084*	
C19	0.1173 (5)	0.1817 (2)	0.37866 (15)	0.0636 (10)	
H19	0.1215	0.2108	0.3451	0.076*	

### Atomic displacement parameters (Å^2^)

**Table:**

	*U*^11^	*U*^22^	*U*^33^	*U*^12^	*U*^13^	*U*^23^
N1	0.0671 (19)	0.0592 (17)	0.0471 (16)	−0.0026 (15)	−0.0064 (14)	−0.0001 (14)
O1	0.0566 (14)	0.0493 (13)	0.0546 (13)	−0.0039 (11)	0.0012 (11)	0.0036 (10)
O2	0.0968 (19)	0.0635 (15)	0.0443 (13)	−0.0053 (14)	−0.0050 (12)	0.0108 (12)
C1	0.051 (2)	0.0505 (19)	0.051 (2)	−0.0018 (16)	−0.0015 (16)	−0.0015 (16)
C2	0.0459 (19)	0.0527 (19)	0.0490 (19)	−0.0018 (15)	−0.0078 (15)	0.0007 (16)
C3	0.0377 (17)	0.0510 (19)	0.0526 (19)	−0.0033 (15)	−0.0039 (14)	0.0078 (16)
C4	0.050 (2)	0.055 (2)	0.051 (2)	−0.0034 (16)	−0.0060 (16)	0.0079 (17)
C5	0.077 (3)	0.058 (2)	0.063 (2)	−0.005 (2)	−0.009 (2)	0.016 (2)
C6	0.086 (3)	0.048 (2)	0.081 (3)	0.0014 (19)	−0.014 (2)	0.006 (2)
C7	0.065 (2)	0.058 (2)	0.064 (2)	0.0025 (18)	−0.0112 (18)	−0.0026 (19)
C8	0.077 (3)	0.070 (3)	0.088 (3)	0.015 (2)	0.017 (2)	0.006 (2)
C9	0.106 (3)	0.088 (3)	0.051 (2)	−0.010 (2)	−0.007 (2)	0.022 (2)
C10	0.062 (2)	0.0515 (19)	0.0428 (18)	−0.0016 (17)	0.0023 (16)	−0.0055 (15)
C11	0.064 (2)	0.076 (2)	0.055 (2)	−0.008 (2)	0.0008 (18)	−0.013 (2)
C12	0.079 (3)	0.074 (3)	0.063 (2)	−0.020 (2)	0.020 (2)	−0.008 (2)
C13	0.091 (3)	0.057 (2)	0.049 (2)	−0.004 (2)	0.014 (2)	0.0008 (17)
C14	0.070 (2)	0.0433 (18)	0.0427 (18)	0.0072 (18)	0.0079 (17)	−0.0064 (15)
C15	0.061 (2)	0.0412 (17)	0.0411 (17)	0.0046 (16)	0.0028 (16)	−0.0054 (14)
C16	0.067 (2)	0.0498 (19)	0.0502 (19)	−0.0001 (18)	−0.0005 (17)	0.0017 (16)
C17	0.065 (2)	0.061 (2)	0.067 (2)	−0.0013 (19)	−0.0048 (19)	0.0021 (19)
C18	0.076 (3)	0.065 (2)	0.068 (3)	0.015 (2)	−0.015 (2)	0.000 (2)
C19	0.088 (3)	0.054 (2)	0.049 (2)	0.016 (2)	−0.004 (2)	0.0052 (17)

### Geometric parameters (Å, °)

**Table:**

N1—C1	1.261 (4)	C9—H9B	0.9600
N1—C10	1.418 (4)	C9—H9C	0.9600
O1—C3	1.379 (4)	C10—C11	1.372 (5)
O1—C8	1.413 (4)	C10—C15	1.420 (4)
O2—C4	1.362 (4)	C11—C12	1.396 (5)
O2—C9	1.418 (4)	C11—H11	0.9300
C1—C2	1.462 (4)	C12—C13	1.352 (5)
C1—H1	0.9300	C12—H12	0.9300
C2—C3	1.385 (4)	C13—C14	1.403 (5)
C2—C7	1.399 (4)	C13—H13	0.9300
C3—C4	1.398 (4)	C14—C19	1.412 (5)
C4—C5	1.378 (5)	C14—C15	1.417 (4)
C5—C6	1.380 (5)	C15—C16	1.407 (4)
C5—H5	0.9300	C16—C17	1.360 (5)
C6—C7	1.367 (5)	C16—H16	0.9300
C6—H6	0.9300	C17—C18	1.396 (5)
C7—H7	0.9300	C17—H17	0.9300
C8—H8A	0.9600	C18—C19	1.350 (5)
C8—H8B	0.9600	C18—H18	0.9300
C8—H8C	0.9600	C19—H19	0.9300
C9—H9A	0.9600		
			
C1—N1—C10	118.3 (3)	O2—C9—H9C	109.5
C3—O1—C8	116.5 (3)	H9A—C9—H9C	109.5
C4—O2—C9	117.8 (3)	H9B—C9—H9C	109.5
N1—C1—C2	122.2 (3)	C11—C10—N1	122.3 (3)
N1—C1—H1	118.9	C11—C10—C15	119.9 (3)
C2—C1—H1	118.9	N1—C10—C15	117.7 (3)
C3—C2—C7	119.1 (3)	C10—C11—C12	120.3 (3)
C3—C2—C1	119.6 (3)	C10—C11—H11	119.8
C7—C2—C1	121.3 (3)	C12—C11—H11	119.8
O1—C3—C2	119.0 (3)	C13—C12—C11	121.1 (4)
O1—C3—C4	120.1 (3)	C13—C12—H12	119.4
C2—C3—C4	120.9 (3)	C11—C12—H12	119.4
O2—C4—C5	125.1 (3)	C12—C13—C14	120.6 (3)
O2—C4—C3	116.0 (3)	C12—C13—H13	119.7
C5—C4—C3	118.9 (3)	C14—C13—H13	119.7
C4—C5—C6	120.3 (3)	C13—C14—C19	122.4 (3)
C4—C5—H5	119.8	C13—C14—C15	119.3 (3)
C6—C5—H5	119.8	C19—C14—C15	118.3 (3)
C7—C6—C5	121.2 (3)	C16—C15—C14	118.7 (3)
C7—C6—H6	119.4	C16—C15—C10	122.4 (3)
C5—C6—H6	119.4	C14—C15—C10	118.8 (3)
C6—C7—C2	119.6 (3)	C17—C16—C15	121.1 (3)
C6—C7—H7	120.2	C17—C16—H16	119.5
C2—C7—H7	120.2	C15—C16—H16	119.5
O1—C8—H8A	109.5	C16—C17—C18	120.0 (4)
O1—C8—H8B	109.5	C16—C17—H17	120.0
H8A—C8—H8B	109.5	C18—C17—H17	120.0
O1—C8—H8C	109.5	C19—C18—C17	120.6 (4)
H8A—C8—H8C	109.5	C19—C18—H18	119.7
H8B—C8—H8C	109.5	C17—C18—H18	119.7
O2—C9—H9A	109.5	C18—C19—C14	121.2 (3)
O2—C9—H9B	109.5	C18—C19—H19	119.4
H9A—C9—H9B	109.5	C14—C19—H19	119.4
			
C10—N1—C1—C2	−177.7 (3)	C1—N1—C10—C15	−130.1 (3)
N1—C1—C2—C3	−162.3 (3)	N1—C10—C11—C12	177.0 (3)
N1—C1—C2—C7	18.7 (5)	C15—C10—C11—C12	−0.9 (5)
C8—O1—C3—C2	109.7 (3)	C10—C11—C12—C13	0.5 (6)
C8—O1—C3—C4	−73.1 (4)	C11—C12—C13—C14	0.1 (6)
C7—C2—C3—O1	175.4 (3)	C12—C13—C14—C19	178.3 (3)
C1—C2—C3—O1	−3.7 (4)	C12—C13—C14—C15	−0.3 (5)
C7—C2—C3—C4	−1.8 (5)	C13—C14—C15—C16	177.8 (3)
C1—C2—C3—C4	179.1 (3)	C19—C14—C15—C16	−0.7 (4)
C9—O2—C4—C5	3.0 (5)	C13—C14—C15—C10	−0.1 (4)
C9—O2—C4—C3	−176.6 (3)	C19—C14—C15—C10	−178.7 (3)
O1—C3—C4—O2	3.7 (4)	C11—C10—C15—C16	−177.2 (3)
C2—C3—C4—O2	−179.2 (3)	N1—C10—C15—C16	4.8 (4)
O1—C3—C4—C5	−175.9 (3)	C11—C10—C15—C14	0.7 (4)
C2—C3—C4—C5	1.2 (5)	N1—C10—C15—C14	−177.3 (3)
O2—C4—C5—C6	−179.1 (3)	C14—C15—C16—C17	−0.4 (5)
C3—C4—C5—C6	0.5 (5)	C10—C15—C16—C17	177.5 (3)
C4—C5—C6—C7	−1.6 (6)	C15—C16—C17—C18	1.0 (5)
C5—C6—C7—C2	1.1 (6)	C16—C17—C18—C19	−0.4 (5)
C3—C2—C7—C6	0.6 (5)	C17—C18—C19—C14	−0.7 (5)
C1—C2—C7—C6	179.7 (3)	C13—C14—C19—C18	−177.2 (3)
C1—N1—C10—C11	51.9 (4)	C15—C14—C19—C18	1.3 (5)

### Hydrogen-bond geometry (Å, °)

**Table:**

*D*—H···*A*	*D*—H	H···*A*	*D*···*A*	*D*—H···*A*
C8—H8C···O1^i^	0.96	2.54	3.232 (4)	129
C8—H8C···O2	0.96	2.43	2.998 (4)	118
C16—H16···N1	0.93	2.52	2.839 (4)	101

Symmetry codes: (i) *x*−1/2, *y*, −*z*+3/2.
